# Association between Dietary Patterns and Cognitive Function among Qatari Adults: A Cross-Sectional Analysis of the Qatar Biobank Study

**DOI:** 10.3390/nu15184053

**Published:** 2023-09-19

**Authors:** Sundus Fituri, Zumin Shi

**Affiliations:** Human Nutrition Department, College of Health Sciences, QU Health, Qatar University, Doha P.O. Box 2713, Qatar; sf1513326@qu.edu.qa

**Keywords:** diet, dietary patterns, a posteriori dietary patterns, factor analysis, rice, cognition, cognitive function, Qatar Biobank

## Abstract

This study aimed to assess the association between dietary patterns and cognitive function among Qatari adults. In a cross-sectional analysis, data on 1000 Qatari adults attending the Qatar Biobank Study (QBB) aged ≥18 years were obtained. Using factor analysis, dietary patterns were constructed based on habitual dietary intake assessed by food frequency questionnaires (FFQs). The mean reaction time (MRT) derived from self-administered touch screen tests was used as an indicator of cognitive function. The association between dietary patterns and MRT was investigated using linear regression. The mean age of the participants was 35.8 (SD 10.3) years, and the mean MRT was 715.3 (SD 204.1) milliseconds. Three dietary patterns were identified. The “traditional” dietary pattern, characterized by high intakes of white rice, mixed dishes and soups/starters possibly high in saturated fat and sodium, was positively associated with MRT. In the multivariable model, comparing the highest to lowest quartiles of the traditional pattern, the regression coefficient for MRT was 50.0 (95% CI 16.9, 83.1; *p* for trend 0.001). There was an effect modification of diabetes and age on the association between the “modern” dietary pattern and MRT. The “convenient” dietary pattern was not associated with cognition. In conclusion, the traditional rice-based dietary pattern may be associated with poor cognitive function.

## 1. Introduction

Dementia is a syndrome or umbrella term for several diseases characterized by deterioration of cognitive function [1]. In 2019, around 55 million people suffered from dementia, and it is rapidly growing into a major public health concern [2]. As population aging increases worldwide, the total number of people living with dementia is expected to rise to 139 million by the year 2050 [2].

Currently, the strongest known risk factor for dementia is age [3]. However, the development of dementia is not necessarily an inevitable result of aging. In fact, a growing body of evidence supports a relationship between dementia and a number of modifiable lifestyle factors commonly associated with other medical conditions [4]. Such risk factors include smoking, physical inactivity and unhealthy dietary patterns. Certain chronic diseases—including diabetes and hypertension—are also related to an increased risk of dementia [5,6]. In Qatar, diabetes and hypertension constitute two of the four main noncommunicable diseases in the country, with prevalences of 17.4% and 16.8%, respectively [7].

The lack of an established cure for dementia gives importance to the reduction of its risk [8]. Recently, there has been an increasing interest in the role of dietary patterns in the prevention of cognitive decline. For example, adherence to the Mediterranean and DASH diets has been associated with better cognitive outcomes [9]. The more recently introduced “MIND” diet—a combination of the two aforementioned diets—shows the strongest associations and is linked to a reduced risk of Alzheimer’s disease [9,10].

The Mediterranean, DASH, and MIND diets are examples of a priori dietary patterns. A priori dietary patterns are based on current evidence of the relationships between defined dietary components and disease [11]. On the other hand, a posteriori dietary patterns are derived independently of existing knowledge [12]. Little is known about the relationship between a posteriori dietary patterns and cognitive function. Furthermore, the majority of the related evidence revolves around Western populations [13,14,15,16,17,18,19]. One prospective study on Japanese elderly found that higher adherence to a dietary pattern characterized by high intakes of vegetables, algae, soybeans and soybean-related products, milk and dairy products combined with low intakes of rice decreased the risk of incident dementia [20]. In Chinese women, a higher score for a “vegetables-fruits” pattern high in vegetables, fruits, soy products and legumes was related to lower odds of cognitive impairment [21]. Similarly, a case-control study in Iran revealed that a healthy dietary pattern rich in fruits, vegetables, legumes and nuts was associated with decreased odds of Alzheimer’s disease [22].

In Qatar, a posteriori dietary patterns have only been studied in relation to asthma [23] and diabetes [24,25]. As for cognitive function, a few studies have assessed the effects of intake of nuts [26] and rice [27], patterns of tea and coffee consumption [28], as well as serum magnesium [29], which was found to have an inverse association. No studies currently exist on the specific relationship between dietary patterns and cognitive function in Qatar. Additionally, healthful diets that have already been explored may be very different from the traditional Qatari diet. Since diets are culture-specific, it is important to ascertain whether there is a pattern unique to Qatari customs that would help protect against cognitive disease.

To address these issues, this study aimed to (1) identify different dietary patterns in the sample population; (2) examine the association between these dietary patterns and cognitive function as indicated by mean reaction time; and (3) assess whether this association is mediated by serum magnesium or chronic diseases such as diabetes and hypertension.

## 2. Materials and Methods

### 2.1. Study Design and Sample

This cross-sectional study analyzed the data of adult subjects recruited by the Qatar Biobank (QBB) study. QBB is an ongoing population-based, long-term cohort study enrolling adult participants aged 18 years and older [30]. Participants must be either Qatari or long-term residents of Qatar who have lived in the country for at least 15 years. The recruitment of the participants occurs via the internet, social media, or through family and friends. Established in 2012, the study aims to collect extensive biological, clinical, and lifestyle information on 60,000 men and women. Up to the end of 2020, more than 26,000 individuals have been recruited into the study [31].

To be recruited, the participants are invited for a health examination at the QBB facility in Hamad Medical City. The examination involves a self-administered questionnaire and a nurse interview. Sociodemographic data, lifestyle factors, and dietary habits are collected through the questionnaire. The nurse interview collects information on medical history, family history of disease, and use of medications. Research nurses also measure body weight and height via a Seca stadiometer. Blood samples of 60 mL are drawn and analyzed for a total of 66 biomarkers at Hamad Medical Corporation.

In the current study, we used a random sample of 1000 Qatari participants (500 men and 500 women) selected from the QBB database. This sample size was chosen due to QBBs provision of free access to the data of 1000 participants for the purpose of student research. The inclusion criteria were based on complete examination data, namely the food frequency questionnaire and the cognitive function test.

### 2.2. Outcome Variable: Mean Reaction Time

Among QBB participants, cognitive function is evaluated based on two tests from the Cambridge Neuropsychological Test Automated Battery (CANTAB): the Reaction Time (RT) test and the Paired Associates Learning (PAL) test [32,33]. The RT test is a self-administered computerized assessment of psychomotor speed, comprising 60 trials. In each trial, a visual target appears as a small white box positioned within either of two larger black boxes. The participant is required to rapidly select the box where the target is presented, which varies in location on the screen. The mean reaction time (MRT) was derived from a calculation of the average time the participants took to complete these trials. Higher MRT equates to lower cognitive function. The PAL test, which assesses paired episodic memory, was not considered in the present study due to the limited variation in its results.

### 2.3. Exposure Variable: Dietary Patterns

Dietary intake of QBB participants is assessed using a self-administered computer-based food frequency questionnaire (FFQ) [30]. The FFQ includes 102 food items that represent dietary habits as the frequency of consumption of different foods and beverages over the previous year [7]. Adapted from the European Prospective Investigation into Cancer and Nutrition (EPIC) study, this FFQ is yet to be validated in the Qatari population. However, the food items of the questionnaire are comparable to those of a recently validated FFQ in Qatar [34]. Furthermore, the FFQ was assessed for its internal validity prior to its use in the survey [30]. Internal validity was determined by examining intake of broad categories of foods (chicken, fish, meat, fast or take-away foods etc.) in relation to the sum of individual food items under these larger categories. Spearman’s rank correlations ranged between moderate for snacks (0.3) and high for fish (0.74) [23].

In the current analysis, the 102 food items were collapsed into groups according to similarities in nutrient composition and/or cooking methods (Supplementary Table S1). For example, cookies, biscuits and cake were classified under the “Desserts” group. The frequency of consumption of these food groups was recoded into times per week. This frequency was used as the input variable for the construction of the dietary patterns using factor analysis. The number of dietary patterns extracted was determined based on the criteria of eigenvalues > 1, the scree plot and the interpretability of the patterns in relation to food culture in Qatar. Varimax rotation was used to aid in the interpretation of the derived dietary patterns. Finally, each participant was given a dietary pattern score based on pattern-specific factor loadings of the constituent food groups and the frequency of their weekly intake of those foods.

### 2.4. Covariates

In the present analysis, the following variables were considered covariates: age, sex, education (low: below university; high: university or above), smoking status (non-smokers, smokers, and ex-smokers), physical activity level (metabolic equivalent of task (MET), recoded as tertiles), and body mass index (BMI). Overweight was defined as a BMI of 25.0–29.9 kg/m^2^; obesity was defined as a BMI ≥ 30 kg/m^2^. Other covariates included diabetes and hypertension, due to their known relationship with cognitive decline and dementia [5,6]. Diabetes was defined as a self-reported diagnosis or based on the following diagnostic criteria: HbA1c ≥ 6.5%, random blood glucose ≥ 200 mg/dL, or fasting blood glucose ≥ 126 mg/dL [35]. Elevated systolic blood pressure (>140 mmHg) and/or diastolic blood pressure (>90 mmHg) or a previous diagnosis of hypertension was used to define hypertension [36]. Seeing as serum magnesium was recently shown to be associated with cognitive function among Qatari adults from the QBB [29], it was also considered a covariate in this analysis.

### 2.5. Statistical Analyses

For the description of the sample characteristics, differences between groups were compared using the chi-square test for categorical variables and ANOVA for continuous variables. Dietary pattern scores were recoded into quartiles. The groups compared were based on quartiles of each identified dietary pattern. The lowest quartiles were used as the reference groups.

To assess the association between the quartiles of dietary patterns and MRT, four multivariable linear regression models were used. Model 1 was adjusted for age and sex. Model 2 was further adjusted for education, smoking and physical activity. Model 3 included additional adjustments for BMI, diabetes, hypertension, and the use of medication for these diseases. The fourth model adjusted for covariates in model 2 in addition to serum magnesium. To assess mediation, structural equation models were constructed to examine the direct and indirect effects (via BMI, blood pressure, blood glucose, hemoglobin A1C (HbA1c), total cholesterol, low-density lipoprotein (LDL), high-density lipoprotein (HDL) and triglycerides) of the dietary patterns on cognitive function. In subgroup analyses, multiplicative interactions between the different dietary patterns and several covariates relative to MRT were explored through the addition of their product terms in the linear regression models. Stata’s marginsplot command was used to visualize the interactions. All of the analyses were performed using STATA 17 (StataCorp). Statistical significance was considered when *p*-values were less than 0.05 (two-sided).

### 2.6. Ethical Considerations 

Under its Ethics Committee, Hamad Medical Corporation’s Institutional Review Board (IRB) approved the Qatar Biobank study in 2011 [7]. All recruited participants provided their written informed consent. The present study was approved under a category of IRB exemption by the QBB.

## 3. Results

### 3.1. Sample Characteristics

Among the participants, the mean age was 35.8 (SD 10.3) years, while the mean MRT was 715.3 (SD 204.1) milliseconds. Most of the participants had a high education (66.1%) and did not smoke (67.3%). Overall, they had low physical activity. The majority of the subjects (70.7%) were either overweight (38.2%) or obese (32.5%), with a mean BMI of 28.2 kg/m^2^ (SD 28.2). A total of 12.1% of participants were diabetic, and 9.6% were hypertensive.

**Figure 1:**
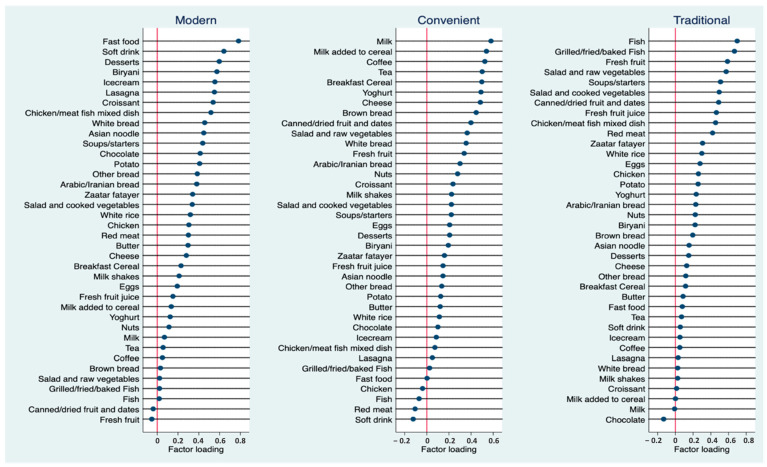
Factor loadings of three identified dietary patterns (Modern, Convenient and Traditional).

Three dietary patterns were identified. Figure 1 shows the factor loadings of the identified dietary patterns. Based on their constituent foods, the patterns were named “modern”, “convenient” and “traditional”. The modern pattern was characterized by foods high in sugar, refined grains, and fat, namely fast food, soft drinks, desserts, biryani, ice cream, lasagna, croissants, mixed dishes, white bread, and Asian noodles. The convenient pattern consisted mainly of “breakfast” and “snack” foods, with high factor loadings of milk, milk added to cereal, coffee, tea, breakfast cereal, yogurt, cheese, brown bread, canned or dried fruits, and dates. The traditional pattern shared several characteristics with the classical prudent diet, emphasizing fish, fresh fruits, vegetables, canned or dried fruits, dates, and fruit juice. The pattern was also heavily loaded with soups and starters, mixed dishes, red meat, fatayer (traditional pastries), and white rice. Together, the three patterns explained 32% of the total variation in dietary intake.

Sample characteristics by quartiles of dietary pattern are shown in Table 1. Participants with high intakes of the modern dietary pattern were more likely to be younger and less educated, with a lower likelihood of having diabetes or hypertension or using medications for these diseases. The convenient dietary pattern revealed no major differences in most sample characteristics across its different levels of intake. Across the quartiles of the traditional dietary pattern, there was an increase in age (Q4 vs. Q1: 38.6 vs. 32.7 years). In addition, BMI increased from 27.8 kg/m^2^ in Q1 to 29.0 kg/m^2^ in Q4. Compared to participants with the lowest intake of the traditional dietary pattern, those with the highest intake were more likely to have diabetes or hypertension (Q4 vs. Q1: 18.7% vs. 9.3% for diabetes; 14.8% vs. 3.6% for hypertension).

### 3.2. Dietary Patterns and Mean Reaction Time

Of the three identified patterns, the traditional diet was positively associated with MRT (Table 2). After adjusting for age and sex, the regression coefficients for MRT were 0.0 (reference), 4.5 (95% CI −27.5, 36.6), 33.9 (95% CI 1.5, 66.2), and 49.2 (95% CI 16.5, 81.9) across the quartiles of traditional pattern scores (*p* for trend 0.001). Further adjustment for lifestyle factors, chronic disease, and medication use had little effect on the association. In the fully adjusted model, comparing the highest to lowest quartiles of the traditional dietary pattern, the regression coefficient for MRT was 50.0 (95% CI 16.9, 83.1; *p* for trend 0.001). The association was independent of serum magnesium (Model 4). In the overall sample, no significant associations between the modern and convenient dietary patterns with cognition were found.

### 3.3. Mediation and Subgroup Analyses

#### 3.3.1. Mediation Effects

No indirect effects of the traditional dietary pattern on MRT via magnesium (Table 3), BMI, systolic blood pressure, diastolic blood pressure, blood glucose, HbA1c, triglycerides, LDL, or HDL were found using structure equation models. The indirect effects of total cholesterol on the association between the traditional dietary pattern and MRT were borderline significant (Table 4).

#### 3.3.2. Effect Modifications

**Figure 2:**
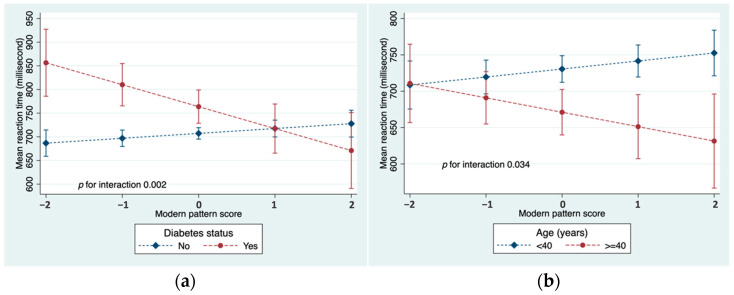
(**a**) Interaction between modern dietary pattern and diabetes in relation to MRT; (**b**) Interaction between modern dietary pattern and age in relation to MRT. Both models adjusted for age, sex, education, smoking, and physical activity.

There was a significant interaction between the modern dietary pattern and diabetes in relation to MRT (Figure 2a). An inverse association between the diet and MRT was observed in those who had diabetes. In addition, there was a significant interaction with age (Figure 2b). Among younger participants who were less than 40 years old, the modern dietary pattern was positively associated with MRT, while older participants tended to show an inverse association. There was no statistical evidence that the association between the modern diet and MRT was modified by sex, education, smoking, physical activity, BMI, blood lipids, or hypertension (Supplementary Table S2). No effect modification was found in the associations between the traditional and convenient patterns with MRT.

### 3.4. Individual Food Intake and Mean Reaction Time

The association between quartiles of individual food intake and MRT is presented in Supplementary Table S3. The individual foods examined were those from the traditional dietary pattern with factor loadings of 0.3 or above. After adjusting for sociodemographic and lifestyle factors, the consumption of soups/starters (Q4 vs. Q1: β 41.0; 95% CI 9.7, 72.3; *p* for trend 0.027), mixed dishes (Q4 vs. Q1: β 58.4; 95% CI 26.3, 90.5; *p* for trend 0.001), and white rice (Q4 vs. Q1: β 39.3; 95% CI 8.5, 70.1; *p* for trend 0.006) were associated with increased MRT. No associations were found with fish, fresh fruits, vegetables, canned/dried fruits (including dates), fruit juice, red meat, or traditional pastries/breads.

### 3.5. Dietary Patterns and Chronic Conditions

The traditional dietary pattern was positively associated with hypertension (Supplementary Table S4). In the multivariable model, the odds ratios for hypertension were 1.00 (reference), 1.56 (95% CI 0.63, 3.86), 2.48 (95% CI 1.05, 5.82) and 2.41 (95% CI 1.03, 5.66) across the quartiles of traditional dietary pattern scores (*p* for trend 0.026). No associations were found between the traditional dietary pattern and obesity or diabetes. The modern and convenient dietary patterns were not related to chronic disease.

## 4. Discussion

In this cross-sectional study of 1000 QBB participants, dietary patterns were constructed using factor analysis to examine their associations with cognitive function. Factor analysis is widely used as a tool to investigate dietary patterns in relation to health outcomes in epidemiological research [12,37]. The traditional dietary pattern, characterized by high intakes of fish, fruits, and vegetables, was positively associated with MRT. Further analyses of individual food groups in this pattern revealed that only soups and starters, mixed dishes, and white rice were related to increased MRT. In the overall sample, there were no associations between the modern dietary pattern and MRT. However, upon subgroup analyses, the modern food pattern was inversely associated with MRT in older participants and in people who had diabetes. No significant associations between the convenient dietary pattern and cognition were found.

Three main dietary patterns were identified in this study, similar to the number extracted in other studies using factor analysis of dietary intake data from FFQs [23,24,38,39,40,41]. In a Qatari population, Shi et al. (2022) identified three dietary patterns: traditional, prudent, and fast food/sweets [23]. The modern dietary pattern from the present study resembles the fast-food/sweets pattern previously identified, while the current traditional pattern shares similarities with both the established prudent and traditional patterns [23,24]. In addition, the modern dietary pattern was more likely to be followed by younger participants, while the traditional pattern became increasingly popular with age, an observation similarly reported by other studies [24,42]. In the current study, the three dietary patterns explained 32% of the total variation in dietary intake, which is comparable to percentages published elsewhere [23,41].

### 4.1. Dietary Patterns and Cognition

#### 4.1.1. Traditional Dietary Pattern

The finding of a positive association between the traditional dietary pattern and cognitive decline is inconsistent with the literature. Similar to healthy dietary patterns—such as the Mediterranean, DASH, and MIND diets—the current traditional pattern consisted of high intakes of fish, fruits, and vegetables, components often linked with improved cognitive outcomes, including reduced risk of dementia [9,43,44]. This association was also demonstrated using data-driven methods. For example, in a 4-year prospective cohort, a dietary pattern (derived through reduced rank regression) characterized by high intakes of salad dressing, nuts, fish, tomatoes, poultry, fruits, cruciferous vegetables and green leafy vegetables was strongly associated with a lower risk of incident Alzheimer’s disease (T3 vs. T1: HR 0.62; 95% CI 0.43, 0.89; *p* for trend 0.01) in a US population of older adults [18]. These findings conflict with those of the present study, which showed a diet high in fish, fruits, and vegetables to worsen cognitive function. However, it is important to note that upon further analysis, increased MRT was found to be attributed to other food groups in the traditional pattern, namely white rice, mixed dishes, and soups/starters.

The link between white rice and cognitive decline has been supported previously. In a cross-sectional study of a Qatari population, higher rice consumption was associated with increased MRT (Q4 vs. Q1: β 34.5; 95% CI 2.6, 66.4; *p* for trend 0.017) after adjusting for age, sex, and lifestyle factors, including fruit and vegetable intake [27]. These results were in agreement with two prospective studies conducted in China. In a study that combined data from the Shanghai Women’s Health Study and the Shanghai Men’s Health Study, higher rice intake was significantly associated with over 20% higher odds of impaired memory and over 45% higher odds of impaired decision-making [45]. Additionally, a 2-year cohort study revealed a direct association between higher weekly rice consumption and an increased risk of mild cognitive impairment (MCI) in Chinese older adults (HR 1.05; 95% CI 1.01, 1.10; *p* 0.019) [46]. By contrast, one cross-sectional study in China concluded that participants in the highest quartile of rice intake had 17% lower odds of MCI [47]. However, the authors noted that an even greater benefit was seen at the moderate intake level of the third quartile (OR 0.65; 95% CI 0.54, 0.78), suggesting a positive effect of rice consumption within a certain range [47]. Furthermore, a crossover trial in Japan reported lower cognition scores in 31 older adults after consuming white rice 3 times a day for 6 months compared to consuming the same amount of brown rice [48]. In the current study, brown rice was not included in the FFQ for the collection of dietary data from the participants, as white rice is more common among the Qatari population [49].

Several mechanisms may explain the association between white rice and MRT found in the current study. For one, white rice is the product of the refining and polishing of the intact rice grain, which strips the outer bran and germ layers along with the fiber, vitamins, and minerals they contain. Dietary fiber and other micronutrients, such as magnesium, that are removed from white rice have been independently associated with improved cognitive outcomes [50,51]. However, in the present study, serum magnesium did not mediate the effects of the traditional dietary pattern on MRT, despite the high factor loading of white rice. Nonetheless, another study of Qatari adults found low serum magnesium to be associated with pre-diabetes and diabetes [25]. Though we did not find a mediating effect of blood glucose or HbA1c on the association between the traditional diet and MRT in the present study, there is growing evidence that diabetes predisposes to cognitive decline [6]. In a large meta-analysis of 144 prospective studies, diabetes was associated with a 25% to 95% higher risk of cognitive impairment and dementia [6]. Previous meta-analyses have concluded that intake of white rice is associated with a higher risk of diabetes, particularly among Asian populations [52,53]. One meta-analysis of cohort studies observed a linear dose-response at intake levels above 300 g/day, with a 13% higher risk of diabetes for every serving increment of 158 g/day, suggestive of a threshold [54]. Furthermore, refined grains such as white rice tend to have a high glycemic index (GI) due to their low fiber content, which leads to a faster rate of absorption [55]. A systematic review suggested that low-GI meals may benefit cognitive performance in some studies; however, overall findings remain inconclusive [56,57].

In the present study, “mixed dishes” were also positively associated with reduced cognitive function. The mixed dishes group represented foods that were collapsed together according to similarities in nutrient composition and/or cooking methods to facilitate factor analysis. The group included combinations of rice with meat, chicken, or vegetables, as well as meat or chicken curries, meat/chicken kofta or kebabs, and traditional foods such as “harees” (Supplementary Table S1). Seeing as most of the mixed dishes contained white rice, it can be inferred that rice-related dietary patterns may be another possible link between the traditional diet and increased MRT in our study. In Qatar, rice is a staple food typically served with high-saturated-fat foods, such as lamb meat [49]. Moreover, data from household expenditure surveys found that lamb meat was the most commonly consumed animal product among Qataris [49]. The popular dish “harees”, which was included in this study’s FFQ, is a blend of lamb meat, wheat, and ghee, another source of saturated fat. Higher saturated fat intake has been shown to increase the risk of cognitive impairment and Alzheimer’s disease [58]. Hence, the traditional diet could be seen as a rice-related eating pattern high in saturated fat foods, which may partially explain its association with lower cognitive function.

On the other hand, our study found an indirect effect of total cholesterol on the association between the traditional dietary pattern and MRT. Interestingly, the pattern was associated with low total cholesterol, which in turn was related to worse cognitive function. Studies on the association between total cholesterol and cognitive performance remain inconclusive. In a study of the Framingham Heart Study cohort, participants with lower total cholesterol performed less well in multiple cognitive domains, such as verbal fluency, abstract reasoning, and attention or concentration [59]. Conversely, another large cohort study showed that subjects with high total cholesterol levels (≥240 mg/dL) had a 42% increased risk of late-life dementia compared to those who had lower levels [60]. The inconsistency in these findings may be explained by both the neuroprotective and aggravating effects of cholesterol in relation to cognition. As part of the cell membrane, cholesterol plays an important role in the maintenance of neuronal function and plasticity; however, it also contributes to the formation and aggregation of amyloid plaques [61]. Moreover, it has been suggested that the association between cholesterol and cognitive function may be strongly age-dependent [62]. A review concluded that mid-life high cholesterol levels are associated with increased risk of late-life dementia and cognitive decline, while measurements taken in late-life may show an inverse association [62]. In the current study, it might be possible that, assuming low serum cholesterol, consumption of the traditional diet exacerbated cognitive dysfunction.

Lastly, another possible mechanism lies in the association between hypertension and cognition. In our study, the traditional dietary pattern was associated with increased odds of hypertension (Supplementary Table S4), though no indirect effects via systolic or diastolic blood pressure were found. Qatari cuisine has been shown to be high in sodium, despite national efforts to lower salt consumption in the country [49,63]. In our study, soups and starters were found to be associated with increased MRT. This food category consisted of high-sodium items, such as instant soups and processed meats, as well as traditional dips (e.g., hummus) that might be prepared with high amounts of salt. High salt intake is a well-established risk factor for hypertension [64]. Hypertension is known to have harmful effects on the brain, including damage to the cerebral vasculature, injury to white matter regions critical for cognitive function, and promotion of Alzheimer’s pathology through increased amyloid plaque and neurofibrillary tangle burden [65]. Furthermore, a study of a large prospective cohort concluded that excessive sodium intake impairs cognitive function independently of hypertension and other known risk factors [66]. Thus, it can be speculated that high sodium intake in the traditional Qatari diet may have played a role in its association with MRT, either directly or indirectly via increased blood pressure.

#### 4.1.2. Modern Dietary Pattern

The effect modification of diabetes and age on the association between the modern dietary pattern and MRT was intriguing. In our study, it was unexpectedly observed that the modern diet was associated with decreased MRT in older and diabetic participants. Characterized by foods high in sugar, refined grains and fat, the modern pattern shares resemblance with a Western-style diet, which has been linked to both diabetes and cognitive decline [67,68]. However, in a study of Qatari adults with type 2 diabetes, the modern dietary pattern was inversely associated with poor glycemic control, though there was a significant interaction with medication use in men [24]. The authors concluded that men who consumed “unhealthy food” were more likely to achieve glycemic control through medication than diet [24]. Hence, the modern pattern and MRT association in our study may be partially explained by glycemic control through antidiabetic treatment in those with the highest scores for the diet. Glycemic control or lower HbA1C levels have been correlated with improved cognitive function in individuals with diabetes [69]. The effect modification by age might be due to the higher likelihood of older individuals having diabetes, possibly well controlled by medications. Due to the small number of diabetic participants in our sample, we were unable to investigate the role of medication use in the association between the modern diet and MRT.

#### 4.1.3. Convenient Dietary Pattern

The null association between the convenient dietary pattern and MRT was somewhat unexpected. The convenient diet consisted of high intakes of dairy products, including milk, yogurt, and cheese. It has previously been suggested that the phospholipids in the milk fat globule membrane may influence cognition through their high content of choline derivatives, which play an important role in the nervous system [70]. For example, the choline derivative sphingomyelin is essential in the development of neuronal myelin sheaths and the production of neurotransmitters in the brain [71]. However, a meta-analysis of three prospective cohorts revealed no associations between milk intake and cognitive function, in line with our current findings [72]. In addition to dairy products, the convenient dietary pattern was heavily loaded with coffee and tea, which have shown mixed results regarding their effect on cognition [73,74,75]. A study of a Qatari population found an inverse association between intake of regular coffee and herbal tea with MRT [28]. Other types of coffee and tea showed no associations. In our study, different types of tea and coffee were grouped into single categories to facilitate factor analysis, which may have diminished their individual effects. Overall, the interactions between food items in the dietary pattern may have played an important role in the null association between the convenient diet and MRT.

### 4.2. Strengths and Limitations

To our knowledge, this is the first study to assess the association between dietary patterns and cognitive function in Qatar. This study has several strengths. Firstly, this study sample was a random selection of participants from QBB, an ongoing cohort of the general Qatari population. Secondly, due to the detailed information available, we were able to adjust for various confounders, such as lifestyle factors and chronic diseases, including diabetes and hypertension. In addition, through subgroup analyses, we were able to examine interactions between different dietary patterns and chronic diseases in relation to cognition. An added strength lies in this study’s FFQ, which consisted of a broad selection of 102 foods commonly consumed in Qatar.

However, this study also has several limitations. For one, the cross-sectional design makes it difficult to establish temporality or causality. Second, this study included a sample size of 1000 participants who were mostly young, which may limit generalization. Third, although our sample was selected at random, the participants were originally enrolled in QBB as volunteers. In addition, the outcome of MRT was used as the only measure of cognition. Other aspects of cognitive function were not further explored, though reaction times have been previously proposed as good predictors of cognitive performance [76]. MRT in itself partially relies on visual acuity, which could not be separated from cognition and may have potentially created confounding. Additionally, other important variables that may affect cognitive function, such as stroke and psychological disorders, were not controlled for in the analysis. Hence, while multivariable models were used, the possibility of residual confounding cannot be fully excluded.

The assessment of dietary intake in this study poses some limitations. The qualitative FFQ used in QBB did not specify portion sizes. Hence, we were not able to obtain data about the amounts of foods consumed or adjust for energy intake. However, it has been suggested that portion sizes in quantitative FFQs add limited information to the variance of food intake, which can mostly be explained by frequency of consumption [77]. Additionally, we adjusted for BMI, which may partially remove the confounding effect of energy intake [78]. As with many dietary assessment tools, another drawback of the FFQ is that it is prone to measurement error due to recall bias [79]. On the other hand, FFQs enable the assessment of usual and longer-term intake, and are useful in ranking individuals in epidemiological research [80]. Furthermore, although the FFQ was pre-tested for internal validity, it has not been externally validated in our study population.

## 5. Conclusions

In conclusion, the traditional dietary pattern was positively associated with mean reaction time. High intakes of white rice, mixed dishes and soups or starters possibly high in saturated fat and sodium may impair cognitive function. These findings are of public health importance, as they highlight the potential impact of the whole diet rather than a single nutrient or food. Healthy dietary patterns should be promoted in the prevention of cognitive decline. However, due to the limitations of this study, the results should be interpreted with caution. Further well-designed and long-term studies are warranted to validate our exploratory findings.

## Figures and Tables

**Table 1:** Sample characteristics by quartiles of modern, convenient and traditional dietary pattern scores ^1^.

	Total	Modern Dietary Pattern			Convenient Dietary Pattern			Traditional Dietary Pattern		
		Q1	Q4	*p*-Value	Q1	Q4	*p*-Value	Q1	Q4	*p*-Value
	*n* = 1000	*n* = 250	*n* = 250		*n* = 250	*n* = 250		*n* = 250	*n* = 250	
Mean reaction time (milliseconds)	715.3 (204.1)	762.9 (255.1)	687.6 (175.1)	<0.001	700.8 (224.2)	730.2 (204.0)	0.45	681.0 (159.4)	759.2 (246.2)	<0.001
Age (years)	35.8 (10.3)	41.5 (11.3)	31.0 (8.2)	<0.001	32.4 (9.6)	37.6 (9.8)	<0.001	32.7 (7.8)	38.6 (11.9)	<0.001
Gender				0.17			0.25			<0.001
Male	500 (50.0%)	118 (47.2%)	119 (47.6%)		138 (55.2%)	117 (46.8%)		95 (38.0%)	137 (54.8%)	
Female	500 (50.0%)	132 (52.8%)	131 (52.4%)		112 (44.8%)	133 (53.2%)		155 (62.0%)	113 (45.2%)	
Education				<0.001			0.34			0.14
Low	338 (33.9%)	71 (28.5%)	111 (44.6%)		95 (38.2%)	79 (31.6%)		94 (37.8%)	83 (33.2%)	
High	660 (66.1%)	9178 (71.5%)	138 (55.4%)		154 (61.8%)	171 (68.4%)		155 (62.2%)	167 (66.8%)	
Smoking status				0.50			0.31			0.71
Non-smokers	673 (67.3%)	179 (71.6%)	161 (64.4%)		170 (68.0%)	165 (66.0%)		168 (67.2%)	161 (64.4%)	
Smokers	187 (18.7%)	39 (15.6%)	54 (21.6%)		42 (16.8%)	45 (18.0%)		45 (18.0%)	49 (19.6%)	
Ex-smokers	140 (14.0%)	32 (12.8%)	35 (14.0%)		38 (15.2%)	40 (16.0%)		37 (14.8%)	40 (16.0%)	
Leisure time physical activity (MET hours/week)	6.3 (22.5)	8.0 (22.7)	7.2 (32.0)	0.26	6.7 (20.7)	6.7 (32.2)	0.64	2.6 (10.6)	11.2 (36.6)	<0.001
BMI (kg/m^2^)	28.2 (5.7)	28.9 (5.4)	28.0 (6.3)	0.13	27.7 (6.3)	29.0 (5.4)	0.086	27.8 (5.8)	29.0 (5.7)	0.034
BMI categories				0.010			0.27			0.015
Normal	293 (29.3%)	54 (21.6%)	85 (34.0%)		84 (33.6%)	58 (23.2%)		86 (34.4%)	57 (22.8%)	
Overweight	382 (38.2%)	106 (42.4%)	82 (32.8%)		91 (36.4%)	100 (40.0%)		86 (34.4%)	107 (42.8%)	
Obese	325 (32.5%)	90 (36.0%)	83 (33.2%)		75 (30.0%)	92 (36.8%)		78 (31.2%)	86 (34.4%)	
Serum magnesium (mmol/L)	0.84 (0.06)	0.84 (0.06)	0.83 (0.06)	0.43	0.84 (0.06)	0.83 (0.06)	0.34	0.84 (0.05)	0.83 (0.07)	0.74
Total cholesterol (mmol/L)	4.9 (0.9)	4.9 (0.9)	4.8 (0.9)	0.003	4.8 (0.9)	5.0 (0.9)	0.23	4.9 (0.9)	4.8 (0.9)	0.12
LDL-cholesterol (mmol/L)	3.0 (0.9)	2.9 (0.8)	2.8 (0.9)	0.002	2.9 (0.8)	3.0 (0.8)	0.58	2.9 (0.8)	2.9 (0.9)	0.015
HDL-cholesterol (mmol/L)	1.4 (0.4)	1.4 (0.4)	1.4 (0.4)	0.26	1.3 (0.3)	1.4 (0.4)	0.061	1.4 (0.4)	1.3 (0.3)	0.001
HbA1c (%)	5.5 (0.9)	5.7 (0.9)	5.4 (0.7)	0.006	5.5 (1.0)	5.7 (1.1)	0.020	5.5 (0.9)	5.8 (1.2)	<0.001
Diabetes	116 (12.1%)	41 (16.8%)	21 (8.9%)	0.050	26 (11.0%)	38 (15.6%)	0.24	22 (9.3%)	45 (18.7%)	<0.001
Hypertension	96 (9.6%)	40 (16.0%)	15 (6.0%)	<0.001	18 (7.2%)	28 (11.2%)	0.46	9 (3.6%)	37 (14.8%)	<0.001
Diabetes medication use (other than insulin)	55 (5.5%)	22 (8.8%)	6 (2.4%)	0.013	14 (5.6%)	19 (7.6%)	0.33	9 (3.6%)	27 (10.8%)	<0.001
Insulin use	19 (1.9%)	6 (2.4%)	4 (1.6%)	0.69	3 (1.2%)	10 (4.0%)	0.040	2 (0.8%)	10 (4.0%)	0.004
Hypertension medication use	55 (5.5%)	25 (10.0%)	9 (3.6%)	0.003	12 (4.8%)	19 (7.6%)	0.42	5 (2.0%)	28 (11.2%)	<0.001

^1^ Data are presented as mean (SD) for continuous measures and n (%) for categorical measures.

**Table 2:** Associations between dietary pattern scores (modeled as quartiles and a continuous variable) and mean reaction time ^1^.

	Q1	Q2	Q3	Q4	*p*-Value	Continuous	*p*-Value
Modern							
Model 1	0.0	−11.2 (−43.6, 21.3)	6.9 (−26.3, 40.2)	11.7 (−22.6, 46.0)	0.333	8.3 (−3.7, 20.3)	0.173
Model 2	0.0	−10.6 (−42.7, 21.5)	7.2 (−25.6, 40.1)	2.2 (−31.8, 36.2)	0.649	4.6 (−7.3, 16.5)	0.449
Model 3	0.0	−10.3 (−42.3, 21.8)	8.0 (−25.1, 41.0)	1.0 (−33.3, 35.4)	0.690	3.4 (−8.6, 15.4)	0.578
Model 4	0.0	−12.5 (−44.6, 19.5)	6.1 (−26.7, 38.9)	−0.3 (−34.3, 33.7)	0.734	3.9 (−8.0, 15.7)	0.525
Convenient							
Model 1	0.0	−23.5 (−55.8, 8.7)	−29.8 (−62.3, 2.6)	−21.3 (−53.9, 11.2)	0.188	−7.5 (−19.0, 4.1)	0.204
Model 2	0.0	−20.0 (−51.9, 11.8)	−25.2 (−57.3, 6.8)	−16.2 (−48.4, 16.0)	0.311	−6.2 (−17.6, 5.2)	0.285
Model 3	0.0	−18.7 (−51.1, 13.8)	−20.4 (−52.9, 12.0)	−14.1 (−46.6, 18.4)	0.415	−5.1 (−16.6, 6.4)	0.386
Model 4	0.0	−19.9 (−51.8, 11.9)	−23.7 (−55.7, 8.3)	−16.5 (−48.6, 15.6)	0.314	−6.2 (−17.6, 5.1)	0.282
Traditional							
Model 1	0.0	4.5 (−27.5, 36.6)	33.9 (1.5, 66.2)	49.2 (16.5, 81.9)	<0.001	14.3 (2.8, 25.9)	0.015
Model 2	0.0	5.4 (−26.3, 37.1)	37.9 (5.7, 70.1)	51.7 (19.1, 84.3)	<0.001	14.6 (3.1, 26.1)	0.013
Model 3	0.0	12.3 (−19.9, 44.5)	37.6 (4.9, 70.4)	50.0 (16.9, 83.1)	0.001	12.4 (0.7, 24.1)	0.038
Model 4	0.0	4.2 (−27.5, 35.9)	37.4 (5.2, 69.5)	50.5 (18.0, 83.1)	<0.001	14.3 (2.8, 25.9)	0.015

^1^ The values are regression coefficients (95% CI) from linear regression. Model 1 was adjusted for age and sex. Model 2 was further adjusted for education, smoking, and physical activity. Model 3 was further adjusted for BMI, hypertension, diabetes, hypertension medication use, and diabetes medication use (including insulin). Model 4 adjusted for covariates in Model 2 in addition to serum magnesium.

**Table 3:** Direct and indirect effects (via serum magnesium) of traditional dietary pattern on mean reaction time ^1^.

	β (95% CI)	*p*-Value
Total effect	14.34 (2.84, 25.83)	0.015
Direct effect	13.96 (2.48, 25.44)	0.017
Indirect effect (via serum magnesium)	0.38 (−0.43, 1.18)	0.356

^1^ Model adjusted for age and gender. Dietary pattern was modeled as a continuous variable in the structure equation model analysis.

**Table 4:** Direct and indirect effects (via total cholesterol) of the traditional dietary pattern on mean reaction time ^1^.

	β (95% CI)	*p*-Value
Total effect	14.34 (2.84, 25.83)	0.015
Direct effect	13.23 (1.75, 24.71)	0.024
Indirect effect (via total cholesterol)	1.11 (−0.16, 2.37)	0.086

^1^ Model adjusted for age and gender. Dietary pattern was modelled as a continuous variable in the structure equation model analysis.

## Data Availability

The data presented in this study may be available on request from Qatar Biobank.

## Supplementary Materials

The following supporting information can be downloaded at: https://www.mdpi.com/article/10.3390/nu15184053/s1, Figure S1: Scree plot of eigenvalues of factors derived from factor analysis; Table S1: Food groups used in factor analysis; Table S2: Subgroup analyses of the association between quartiles of modern dietary pattern scores and MRT; Table S3: Associations between intake of individual traditional pattern foods and mean reaction time; Table S4: Associations between quartiles of traditional dietary pattern scores and hypertension.
