# Supplementary material for: Association between Dietary Patterns and Cognitive Function among Qatari Adults: A Cross-Sectional Analysis of the Qatar Biobank Study
Source: Nutrients. 2023 Sep 19;15(18):4053. doi: 10.3390/nu15184053 (PMC10537779; doi:10.3390/nu15184053)
Supplement: Supplementary file 1 [file nutrients-15-04053-s001.zip › nutrients-2605260-supplementary.pdf]

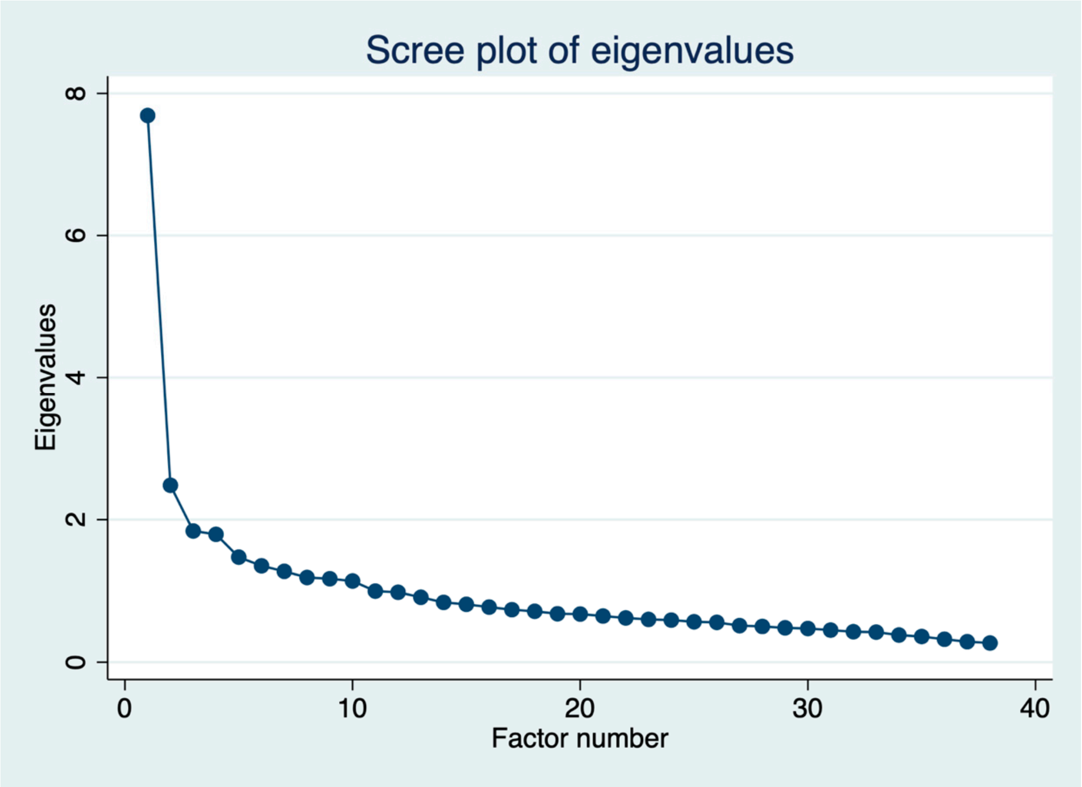

Supplementary Figure S1: Scree plot of eigenvalues of factors derived from factor analysis.

Supplementary Table S1. Food groups used in factor analysis.

Food group	Food items included
Coffee	Arabic coffee; instant coffee; other coffee (e.g. filter coffee, cappuccino)
Tea	Tea; herbal tea; karak
Milk	Milk as a beverage (cold milk, cappuccino)
Milk added to cereal	Milk added to cereal
Milk shakes	Milk shakes or floured milk
Yoghurt	Laban; yoghurt; labneh
Cheese	Hard cheese (e.g. gouda, cheddar); white cheese (halloumi, cream cheese); processed cheese
Butter	Butter
Breakfast Cereal	Balaleet; cornflakes and other cold cereals; muesli, porridge; other brand cereals
Arabic/Iranian bread	Arabic bread (lavash); Iranian bread
White bread	Toast (sliced bread, white)
Brown bread	Toast (sliced bread, brown)
Other bread	Other breads
Croissant	Croissant
Zaatar fatayer	Zaatar fatayer
Soups/starters	Ready-made soup from powder; other soups; hummus, mutabbal, baba ghanush; other dips; tabbouleh; koussa mahshi (stuffed zucchini); eggplants fried or baked; moussaka; other vegetable starters; fish and seafood starters; chicken, meat, or sausage starters
Salad and raw vegetables	Raw vegetables; green leafy salad (e.g. lettuce); mixed salad (e.g. with tomato, onion, cucumber, other vegetables); fattoush; bean salads (chickpeas, lentils); bulghur salad
Salad and cooked vegetables	Vegetable stews; vegetable curries; other cooked vegetables; corn
Potato	Potato
White rice	White rice
Biryani	Biryani
Asian noodles	Asian noodles
Lasagna	Lasagna
Red meat	Red meat
Chicken	Chicken
Fish	Fish
Chicken/meat fish mixed dish	Meat served with rice (mashboos); chicken with rice; harees; meat cooked with vegetables (margooga, saloona); chicken

	cooked with vegetables (margooga, saloona); meat curry; chicken curry; kofta; meat kebab; chicken kebab; lamb chops, escalope; grilled chicken
Grilled/fried/baked fish	Grilled, fried or baked fish; fish cooked with vegetables; smoked fish
Eggs	Eggs (boiled, fried, scrambled, omelets)
Fast food	Meat shawarma; chicken shawarma; falafel wrap; samosa; other Middle Eastern style fast foods; French fries; potato chips; burgers; hotdogs; pizza; fried chicken (e.g. wings, nuggets)
Fresh fruit	Fresh fruit; banana; watermelon
Canned/dried fruit and dates	Canned fruits; dried fruits; dates
Desserts	Traditional desserts (muhalabiyya, halwa, ageeli, elgaymat, khanfaroush); other Middle Eastern or Lebanese desserts; cookies; biscuits; muffins; cake; doughnuts
Chocolate	Chocolate
Ice cream	Ice cream
Nuts	Nuts
Fresh fruit juice	Fresh fruit juices; smoothies
Soft drink	Preserved fruit juice (canned/bottled); soft drinks, sodas; diet soft drinks, sodas; energy drinks

Supplementary Table S2. Subgroup analyses of the association between quartiles of modern dietary pattern scores and MRT.

	Q1	Q2	Q3	Q4	p for trend	p for interaction
Sex						0.534
Male	0.0	-31.4 (-68.2,5.4)	-7.3 (-45.8,31.2)	-14.3 (-54.4,25.9)	0.860	
Female	0.0	10.3 (-42.2,62.8)	27.9 (-24.9,80.7)	25.2 (-29.3,79.8)	0.301	
Age (years)						0.004
<40	0.0	7.2 (-29.1,43.4)	45.9 (10.8,81.0)	27.1 (-7.5,61.7)	0.044	
40+	0.0	-9.9 (-69.5,49.7)	-43.2 (-111.3,24.8)	-34.5 (-118.4,49.3)	0.221	
Education						0.119
Low (below university)	0.0	-38.2 (-112.2,35.7)	-12.6 (-87.2,62.0)	-11.6 (-82.7,59.4)	0.997	
High (university or above)	0.0	-2.5 (-34.7,29.6)	10.8 (-22.3,43.9)	2.2 (-34.0,38.4)	0.702	
Smoking status						0.244
Non-smokers	0.0	-5.8 (-47.2,35.6)	9.2 (-32.6,51.0)	8.6 (-35.5,52.8)	0.573	
Smokers	0.0	-52.7 (-120.9,15.6)	-16.8 (-89.1,55.4)	-55.0 (-126.8,16.9)	0.294	
Ex-smokers	0.0	37.1 (-28.3,102.5)	53.2 (-14.9,121.4)	75.2 (5.0,145.4)	0.034	
Leisure time physical activity						0.330
No	0.0	26.6 (-30.2,83.4)	29.0 (-26.9,85.0)	27.4 (-31.0,85.9)	0.353	
Yes	0.0	-21.0 (-58.8,16.7)	1.7 (-37.4,40.8)	-5.2 (-45.5,35.1)	0.889	
BMI categories						0.055
Normal	0.0	-35.8 (-89.7,18.1)	-10.7 (-62.5,41.1)	-2.9 (-56.0,50.2)	0.722	
Overweight	0.0	11.8 (-34.6,58.1)	48.1 (-1.5,97.8)	23.6 (-28.2,75.4)	0.179	
Obese	0.0	-21.7 (-88.6,45.2)	-24.9 (-93.9,44.1)	-14.9 (-87.7,58.0)	0.671	
HDL cholesterol						0.964
Tertile 1	0.0	-42.6 (-99.8,14.7)	-11.6 (-71.8,48.6)	-6.8 (-63.0,49.4)	0.848	
Tertile 2	0.0	-28.1 (-83.2,26.9)	5.1 (-48.3,58.6)	-2.6 (-60.4,55.2)	0.729	
Tertile 3	0.0	35.3 (-19.5,90.1)	33.4 (-24.2,90.9)	-9.5 (-74.8,55.9)	0.934	
LDL cholesterol						0.323
Tertile 1	0.0	-45.3 (-107.8,17.1)	-9.0 (-73.0,55.1)	-31.0 (-93.2,31.2)	0.581	
Tertile 2	0.0	-13.1 (-72.7,46.5)	-2.3 (-60.3,55.7)	14.9 (-47.1,76.9)	0.569	
Tertile 3	0.0	29.8 (-17.2,76.9)	37.5 (-12.9,88.0)	19.4 (-35.5,74.4)	0.399	

	Q1	Q2	Q3	Q4	p for trend	p for interaction
HDL cholesterol						0.285
Tertile 1	0.0	-35.5 (-91.5,20.4)	-18.9 (-78.1,40.2)	-18.9 (-79.6,41.9)	0.716	
Tertile 2	0.0	-7.8 (-56.8,41.1)	15.7 (-36.9,68.4)	0.4 (-53.1,54.0)	0.804	
Tertile 3	0.0	23.8 (-37.4,85.1)	29.2 (-29.9,88.3)	27.5 (-34.8,89.9)	0.385	
Diabetes						<0.001
No	0.0	9.0 (-21.9,39.9)	31.6 (-0.2,63.4)	23.0 (-9.9,55.9)	0.085	
Yes	0.0	-108.8 (-250.4,32.7)	-126.9 (-278.4,24.6)	-174.1 (-333.1,-15.0)	0.030	
Hypertension						0.238
No	0.0	-12.7 (-43.1,17.7)	8.0 (-22.8,38.7)	3.2 (-28.7,35.1)	0.533	
Yes	0.0	77.5 (-74.8,229.8)	-23.4 (-200.7,154.0)	-12.3 (-196.6,172.1)	0.808	
Insulin use						0.090
No	0.0	-5.7 (-37.9,26.5)	11.2 (-21.6,44.1)	5.5 (-28.6,39.6)	0.542	
Yes	0.0	-316.1 (-799.4,167.3)	21.9 (-606.4,650.1)	-229.5 (-677.1,218.0)	0.350	
Diabetes medication other than insulin						0.105
No	0.0	-4.5 (-35.8,26.8)	12.5 (-19.3,44.4)	4.7 (-28.1,37.4)	0.558	
Yes	0.0	-53.1 (-271.6,165.5)	-64.9 (-330.1,200.4)	-70.3 (-392.6,252.1)	0.585	
Hypertension medication use						0.312
No	0.0	-12.8 (-42.9,17.2)	11.4 (-19.1,41.9)	-1.8 (-33.6,29.9)	0.694	
Yes	0.0	167.3 (-57.5,392.1)	-46.7 (-314.6,221.2)	113.3 (-167.3,394.0)	0.638	

Models adjusted for age, sex, education, smoking and physical activity. Stratification variables were not adjusted in the corresponding models.

Supplementary Table S3. Associations between intake of individual traditional pattern foods and mean reaction time.

	Q1	Q2	Q3	Q4	p for trend
Fish	0.0	-2.0 (-33.7,29.6)	5.0 (-25.1,35.0)	19.2 (-13.8,52.2)	0.254
Fresh fruit	0.0	18.5 (-13.0,50.0)	9.4 (-21.7,40.6)	18.5 (-14.1,51.2)	0.365
Vegetables	0.0	-5.9 (-37.4,25.7)	-1.2 (-32.9,30.5)	21.9 (-10.2,53.9)	0.180
Soups/starters[a]	0.0	2.1 (-29.0,33.2)	-7.0 (-38.3,24.2)	**41.0 (9.7,72.3)**	**0.027**
Canned/dried fruit	0.0	**36.2 (4.1,68.2)**	25.2 (-6.6,56.9)	27.1 (-5.7,60.0)	0.179
Fruit juice	0.0	28.1 (-0.9,57.1)	12.2 (-19.2,43.6)	2.0 (-29.8,33.9)	0.885
Mixed dish[b]	0.0	-2.1 (-33.0,28.8)	-0.0 (-30.5,30.5)	**58.4 (26.3,90.5)**	**0.001**
Red meat	0.0	-0.2 (-25.7,25.3)	9.2 (-24.1,42.5)	-	0.579
Traditional pastries/breads[c]	0.0	-15.5 (-46.1,15.2)	21.7 (-8.9,52.2)	16.8 (-16.0,49.5)	0.097
Rice	0.0	24.0 (-6.1,54.2)	**38.4 (7.3,69.4)**	**39.3 (8.5,70.1)**	**0.006**

Bold represents p<0.05.

Values are regression coefficients (95% CI) from linear regression.

Individual foods are traditional pattern foods with factor loadings ≥ 0.3.

Models adjusted for age, sex, education, smoking and physical activity.

[a] Ready-made soup from powder; other soups; hummus, mutabbal, baba ghanush; other dips; tabbouleh; koussa mahshi (stuffed zucchini); eggplants fried or baked; moussaka; other vegetable starters; fish and seafood starters; chicken, meat, or sausage starters.

[b] Meat served with rice (mashboos); chicken with rice; harees; meat cooked with vegetables (margooga, saloona); chicken cooked with vegetables (margooga, saloona); meat curry; chicken curry; kofta; meat kebab; chicken kebab; lamb chops, escalope; grilled chicken.

[c] Zaatar fatayer; Arabic/Iranian bread.

Supplementary Table S4. Associations between quartiles of traditional dietary pattern scores and hypertension.

	Q1	Q2	Q3	Q4	p-value
Model 1	1.00	1.34 (0.56, 3.17)	2.26 (1.01, 5.05)	1.96 (0.87, 4.42)	0.059
Model 2	1.00	1.56 (0.63, 3.86)	2.48 (1.05, 5.82)	2.41 (1.03, 5.66)	0.026

Values are odds ratios from logistic regression.

Model 1 adjusted for age and sex.

Model 2 further adjusted for education, smoking and physical activity and BMI.
